# Analysis of Acute Non-specific Back Pain Content on TikTok: An Exploratory Study

**DOI:** 10.7759/cureus.21404

**Published:** 2022-01-19

**Authors:** Andrey Zheluk, Judith Anderson, Sarah Dineen-Griffin

**Affiliations:** 1 School of Nursing, Paramedicine and Healthcare Sciences, Charles Sturt University, Bathurst, AUS

**Keywords:** research methods and design, public health informatics, tiktok, social media analytics, lower back pain (lbp)

## Abstract

Introduction

In this study, we evaluated the scope of acute non-specific back pain (ANSBP) content available on TikTok (ByteDance Ltd, Beijing, China) in 2021. It is plausible that TikTok’s popularity among teenagers, adolescents, and young adults may influence decision-making about what constitutes appropriate ANSBP self-care among a younger age cohort.

Methods

We examined 157 of the most viewed videos available through the hashtag #backpain available on TikTok in September 2021. We examined the following research questions: (1) What are the metadata characteristics of the videos in the final data set?, (2) What are the creator identities reflected in the final data set in this study?, (3) What are the ANSBP self-care content themes in the final data set?, and (4) What are the characteristics of the data set based on a low back pain reference checklist based on consensus guidelines?.

Results

We identified clear differences based on TikTok creator identity in our data set of most popular videos. We examined videos created by chiropractors, fitness professionals, influencers, physicians, physiotherapists, and other creator identities. We found that the TikTok videos created by chiropractors were consistently among the most viewed, most commented, and most shared. Conversely, chiropractic TikTok videos consistently had the lowest self-care reference checklist scores relative to all other disciplines. That is, TikTok videos created by chiropractors were least likely to reflect the scientific consensus on treating ANSBP.

Discussion

TikTok is an increasingly popular medium for disseminating short health messages. The main cohort using TikTok is young and at risk of ANSBP. However, we postulate that the messages reaching young TikTok users overall do not generally reflect the self-care advice described in consensus guidelines.

Conclusion

TikTok is a popular social media channel among young people. However, the most viewed TikTok videos about ANSBP are not produced by mainstream health professionals and the videos featuring the #backpain hashtag do not generally reflect contemporary evidence-based practice. There is considerable scope for mainstream health professionals to provide evidence-informed self-management and self-care content for ANSBP on TikTok.

## Introduction

This exploratory study aims to increase the understanding of acute non-specific back pain (ANSBP) content available on TikTok (ByteDance Ltd, Beijing, China). This paper will also examine whether approaches used to analyse YouTube (Google LLC, Mountain View, California, United States) health videos can be applied to TikTok health content. Low back pain imposes a high social and financial burden in all countries across the globe [1]. In the introduction, we have examined the epidemiology of low back pain in younger populations, self-management and self-care of back pain, an overview of TikTok, and approaches to the analysis of health information on TikTok.

Epidemiology of ANSBP in younger populations

Acute low back pain is generally regarded as lasting less than six weeks [2]. In most cases, acute low back pain is non-specific [3,4]. The diagnosis of non-specific low back pain implies no known causes such as infection, tumour, fracture, aneurysm, or cauda equina [2]. ANSBP is also a leading cause of disability among teenagers, adolescents and young adults. Further, low back pain in adolescents and young adults is a significant risk factor for low back pain in adulthood [5,6]. Although ANSBP is common among younger people, it is usually not serious, and usually resolves within a few weeks.

Self-management and self-care of low back pain

Most people manage their low back pain with minimal assistance from health care providers. Independent management without assistance from health care providers is generally referred to as self-care [6]. By contrast, self-management implies health provider-guided patient management of a health condition such as back pain [7]. Researchers have suggested self-management and self-care involve active patient decision making, symptom monitoring, goal setting, and information searching [8]. There are features of social media platforms that may influence patient self-care decision making. For example, researchers have suggested anecdotal information on social media, including the number of views, comments, and likes may influence health decision making. However, indicators such as the number of views, comments, and likes of a TikTok video are not direct measures of influence on health decision making. Rather, these indicators represent video popularity [9-11]. As a consequence, it is plausible that TikTok’s popularity may influence decision making about what constitutes appropriate ANSBP self-care among a younger age cohort. In this study, we examined the most viewed TikTok health videos. These most-viewed TikTok videos may influence self-care decision making about ANSBP among TikTok users. Examination of self-care decision making about ANSBP among TikTok users is beyond the scope of this study.

About TikTok

TikTok is a video-sharing focused social networking service launched in 2018 [12,13]. TikTok hosts a variety of short videos, in fixed categories, including dance and fashion. The duration of these videos ranges from 15 seconds to 3 minutes. Since 2018, TikTok use has grown rapidly. In December 2018, TikTok globally had more than 271 million active monthly users, and 1 billion by September 2021 [14]. A 2021 national United States (US) Pew Research study found phone application users aged 18 to 24 years were likely to report using Instagram (76%), Snapchat (75%), or TikTok (55%) [15]. In 2020, 69% of the TikTok user base were aged 13 to 24 years [16]. In summary, TikTok is a popular social medium used by a younger age cohort. This age cohort is also likely to experience ANSBP.

Health information on TikTok

Google Scholar suggests scientific engagement with TikTok began in 2018. In 2021, health content on TikTok had still not been widely examined in scientific literature. In 2021, there was nascent literature on analysing TikTok videos for health self-care. Among the health care conditions researched were COVID-19 [17], diabetes [18], mental health [19], safe sex [20], dermatology [21], chronic obstructive pulmonary disease (COPD) [22], and oral health [23]. We did not identify any peer-reviewed papers examining TikTok video use for ANSBP.

TikTok has been associated with misinformation in the mass media. In 2021, media monitoring organisation NewsGuard reported COVID-19 vaccine misinformation directed at children aged under 13 on TikTok [24]. The creators suggested TikTok was not adequately monitoring health disinformation. Conversely, TikTok community guidelines are directed at preventing health disinformation [25]. TikTok has reported that community guidelines for creators, including health content, are strictly enforced [26]. 

Approaches to TikTok Health Video Analysis

TikTok in 2021 was a relatively novel medium for health content. The limited peer-reviewed health literature on TikTok in 2021 revealed heterogeneous approaches to research into this social media platform. However, by 2021, there was a larger body of research describing the use of YouTube videos to reach specific groups with condition-specific health messages. In 2021, YouTube was the second most popular social medium and the most popular video social medium [27]. The approaches to analyses of YouTube videos are more mature than that of TikTok. Given the similarities between YouTube and TikTok, we suggest that the methods of analysis of YouTube videos may be applicable to that of TikTok.

Several investigators have examined the methods used to analyse YouTube health content. Drozd and colleagues noted that most researchers develop independent scoring systems and that no commonly agreed on methods exist [28]. Systematic reviews of YouTube analysis methods have focused on the use of validated reference checklists and described the range of approaches and indicators across multiple studies [29,30]. In 2020, Zheluk and Maddock described three broad approaches to the analysis of YouTube health content [31]. These approaches were: (1) analysis of the video metadata exclusive of the content; the most common metadata elements included the number of views, video length, likes, date posted, and language of the video, (2) information quality of videos using validated instruments such as DISCERN (no abbreviation), Journal of the American Medical Association (JAMA) Benchmark Criteria, and Health on the Net Foundation Code of Conduct (HONcode) to evaluate the quality of health information directed at consumers and clinicians; Lee and colleagues reviewed several approaches to assessing health information quality and among the common dimensions by these researchers were accuracy, completeness, consistency, timeliness, validity, and uniqueness [32], and (3) evaluation of video content by expert clinicians by comparing content to reference standards.

Researchers have adopted the methods used for analysing YouTube videos for analysing TikTok health content. These approaches include analysis of information quality using the DISCERN instrument, by creator professional background, by expert clinical review, or by metadata (for example, the number of views, number of comments and likes) [18,21,33]. This research study aims to increase the understanding of ANSBP content on TikTok and to further develop methods for analysing TikTok video content. 

## Materials and methods

This exploratory study aimed to evaluate the scope of ANSBP content available on TikTok in September 2021. In order to evaluate the scope of ANSBP TikTok content, we examined the following research questions (RQs). RQ1: What are the metadata characteristics of the videos in the final data set?, RQ2: What are the creator identities reflected in the final data set in this study?, RQ3: What are the ANSBP self-care content themes in the final data set?, and RQ4: What are the characteristics of the data set based on a low back pain reference checklist?.

We modified the methods developed by Zheluk and Maddock for their analysis of YouTube videos about acute non-specific low back pain (ALBP) [31]. First, in this study, we have assumed that ANSBP TikTok videos are viewed by patients for self-care guidance only (i.e., TikTok videos are viewed independent of health advice). The study by Zheluk and Maddock primarily focused on self-management guidance (i.e., videos intended to complement advice from health and fitness professionals). Second, we have modified the definition of ALBP used by Zheluk and Maddock to include all spinal pain. TikTok videos tagged with #backpain were included in our data set and, thus, include all spinal pain including neck pain. We have expanded the scope of included TikTok videos, as the result of limited anatomical localisation of the pain site in most videos reviewed in the final dataset. Third, in this project, we did not analyse information quality. Zheluk and Maddock found that the Brief DISCERN information quality instrument was less sensitive in identifying differences in the self-management content of YouTube videos than an evidence-based reference list [31]. Similarly, Azer and colleagues suggested that information quality tools were not suitable for analysis of YouTube video content [34].

Data collection and cleansing to obtain the final data set

We first identified ANSBP TikTok videos for analysis through three steps.

Step 1: Selection of Search Terms for Content Discovery

Users can access TikTok videos through the TikTok algorithm or hashtags (#). The TikTok algorithm will serve individualised content to each TikTok user [35]. Hashtags are a facilitator for discovery of specific themed content on TikTok. A single TikTok video may have multiple hashtags. We identified the hashtag “#backpain” as the most viewed relevant TikTok search term (Table 1). By September 2021, TikTok videos with #backpain had produced 1.3 billion views [36]. From a temporal perspective, the views on TikTok represent the aggregate global views of each individual #backpain TikTok video since publication. No national or subnational TikTok view data is publicly available

**Table 1:** Comparative views on TikTok for selected health-related hashtags as of September 30, 2021

TikTok hashtag (#)	Views as of September 30, 2021
#Backpain	1.3 billion views
#Backpainexercises	5.9 million views
#Backpainrelief	236.8 million views
#Chiropractic	3.8 billion views
#Diabetes	2.7 billion views
#Lowbackpain	347.2 million views
#Lowerbackpain	121.6 million views
#Physicaltherapy	1.1 billion views
#Physiotherapy	418.4 million views
#Stroke	499.1 million views
#Yoga	9.2 billion views

Step 2: Raw Data Set

We initially identified a raw data set of the 200 most viewed TikTok videos by searching for #backpain in the TikTok app on September 30, 2021. These 200 TikTok videos were our raw data set, which represented approximately 47% of all TikTok views for #backpain as of September 2021 [36]. Second, we downloaded the metadata for these TikTok videos. We used the TikTok scraper and downloader tool (TSDT) version 1.4.36 in order to scrape the metadata for #backpain TikTok videos in the raw data set [37]. Data scraping is the process of importing data from a website into a spreadsheet for further analysis [38] . The TSDT allows for downloading of metadata and video content for specified number of videos for a specific TikTok hashtag. The relevant fields contained in the metadata include the number of views of each video at the specified date, length, internet address, publisher, and date of publication.

Step 3: Cleansing the Raw Data to Produce the Final Data Set

Of the 200 TikTok videos identified, 43 non-English, duplicate, and not relevant TikTok videos unrelated to back or spinal pain were excluded. The final data set consisted of 157 TikTok videos in English that were relevant to ANSBP. Relevant videos were those that featured spinal pain as the primary theme. The final data set represented approximately 46% of all TikTok videos tagged with #backpain as of September 2021.

Research Questions

We used the final data set in order to answer four RQs.

RQ1: What are the Metadata Characteristics of Videos in the Final Data Set?

We used the metadata obtained via the TSDT to answer this question. Zheluk and Maddock used descriptive statistics to examine length of YouTube videos, the number of views, and channel names [31]. In this study, we used the following data fields: days since published to September 30, 2021, views (PlayCount), video duration, likes (DiggCount), shares, and comments. Through this approach, we were able to describe the metadata characteristics of the final data set.

RQ2: What are the TikTok Creator Identities in the Final Data Set?

We coded the 157 unique TikTok videos in the final data set according to the creator’s identity. We used the same author identities as Zheluk and Maddock did in their analysis of back pain YouTube videos [31]. We used the following six creator identities: chiropractor, fitness professional, influencer, physician, physiotherapist, and other. See Table 2 for creator identity definitions. Researchers have suggested that the creator’s identity contributes to user assessments of source credibility online [39].

**Table 2:** Definition of creator identities

Creator identity		Definition
Chiropractor		Self-identifies as chiropractor OR student of this discipline in TikTok video OR creator identity obtained through Internet search
Fitness		Self-identifies as fitness professional therapist in TikTok video OR creator identity obtained through Internet search
Influencer		No professional or commercial affiliation explicitly listed.
Physician		Self-identifies as medical /osteopathic physician OR student of this discipline in TikTok video OR creator identity obtained through Internet search. Note: US physicians may obtain medical registration through an osteopathic educational pathway.
Physiotherapy		Self-identifies as physiotherapist/physical therapist OR student of this discipline in TikTok video OR creator identity obtained through Internet search
Other		Commercial or other organisational affiliation listed explicitly in TikTok video OR creator identity obtained through Internet search. This includes religious healers, massage providers, yoga, sales, and education providers.

RQ3: What are the Intervention Themes in the Final Data Set?

We first coded each TikTok video according to one of three intervention themes: education, real-time exercise, or real-time treatment. See Table 3 for definitions of intervention themes. These intervention themes were used by Zheluk and Maddock to assess ALBP YouTube content [31]. Second, we analysed the three intervention themes according to creator identities. The six creator identities were: chiropractor, fitness professional, influencer, physician, physiotherapist, and other. 

**Table 3:** Definitions of intervention themes

Intervention	Definition
Education	More than 50% (i.e., the majority of the video) is dedicated to discussion of back pain content or associated aggravating or ameliorating factors including physical and mental health considerations
Exercise	More than 50% (i.e., the majority of the video) is dedicated to real time exercise by clinician or other identity
Treatment	More than 50% (i.e., the majority of the video) is dedicated to real-time hands-on treatment by clinician or other identity on another person, that does not involve exercise or education.

RQ4: What are the Characteristics of the Final Data Set Based on a Reference Checklist?

We analysed the final data set based on the ALBP reference checklist originally developed for the analysis of YouTube videos by Zheluk and Maddock [31]. See Table 4 for the reference checklist codebook. The YouTube checklist was based on recommended first-line ALBP items described by Foster et al. [40]. The checklist included items that an individual patient may reasonably be expected to independently implement as part of a self-management or self-care intervention for ALBP. For the purposes of this TikTok project, we have assumed ALBP self-care guidance is also suitable for ANSBP.

**Table 4:** Acute non-specific back pain self-care reference checklist ANSBP: acute non-specific back pain, ADL: activities of daily living

ANSBP Checklist	ANSBP codebook - definitions
Acute (Suitable for acute patients) Exercise therapy [40]	YES: The exercises are of appropriate intensity and scope for recent-onset ANSBP in a person aged under 40 of average fitness and flexibility level. There are no superior exercises - yoga, pilates, and walking are all equally effective for example.
	NO: The exercises are not suitable as described above.
ADL (Activities of daily living) “Advice to remain active” [40]	YES: At least ONE mention of ADL via narration, desk or visual images. This includes walking, modifying the environment, sport and recreation, modifying ADL, maintaining ADL.
	NO: No mention of ADL as described above
Analgesia Superficial heat NSAIDs [40]	YES: At least one mention of: (1) OTC pharmacological analgesia OR (2) non-pharmacological self-management described in guidelines (for example NSAIDs, heat, ice, pacing, ergonomics)
	NO: No mentions of pharmacological or non-pharmacological analgesia; use of spinal manipulation.
Soothing affect CBT/mindfulness-based stress reduction education [40]	YES: Is the video emotionally soothing overall? This includes education content, editing, music, encouraging fear or catastrophizing, loud or frantic narration
	NO: Not soothing overall as per the description of the soothing affect as described above.
Appropriate Prognosis Education [40]	YES: Author describes: (1) Plausible prognosis consistent with ANSBP guidelines described by Foster et al. [40]. Primarily not overpromising.
	NO: (1) Overpromising. Terms such as “fix”, “cure”, and “instant” indicate unrealistic prognosis. OR (2) No prognosis mentioned.
Red flags	YES: At least one mention of referral to mainstream medical provider (medical practitioner, physiotherapist) for formal diagnosis and advice as a cautionary principle OR Mention of differential diagnoses or symptoms including cauda equina, renal problems, abdominal aortic aneurysm, cancer, infection, and fractures.
	NO: No mention of referral to mainstream medical provider (medical practitioner, physiotherapist) OR No mention of mainstream medical diagnosis for back pain

Intercoder reliability

Coding was conducted by the three authors of this study. Intercoder reliability was achieved through intercoder consensus [41,42]. Following initial coding by Author A, we conducted team-based coding with Authors B and C. We identified an initial discrepancy of 20 individual items within the 157 TikTok videos in the final data set. Following team negotiations, changes to TikTok coding and changes to the codebook were introduced. The final data set and codes represent a team consensus position. No Cohen’s Kappa or Krippendof Alpha tests were performed. In summary, by examining the characteristics of individual TikTok videos, we aimed to determine the relative concordance of TikTok videos produced by different creator identities with the consensus-based low back pain self-management guidelines. 

## Results

We identified clear differences based on the creator’s identity in the TikTok final data set. We found that the TikTok videos created by chiropractors were consistently among the most viewed, most commented and most shared. Conversely, chiropractic TikTok videos generally had the lowest self-care reference checklist scores relative to all other disciplines. That is, TikTok videos created by chiropractors were least concordant with the reference checklist and, thus, these were least likely to reflect scientific consensus on treating ANSBP.

Metadata characteristics of the videos in the final data set

We examined six different metadata characteristics (Table 5). We found on average (mean), chiropractic TikTok videos had the most views (4,744,164); shortest duration (19.97 seconds), were the oldest (317 days), had the most likes, the most shares, and the most comments. The least popular TikTok videos were produced by physiotherapists. Physiotherapy TikTok videos were least viewed (1,583,240), least liked, least shared, and had the fewest comments. The longest videos were created by physicians (mean of 57 seconds).

**Table 5:** Characteristics of TikTok videos according to creator identities

Creator identity	Days since published (Calculated November 8, 2021)	Views	Duration video in seconds	Likes	Shares	Comments
Chiropractic	317	4,744,164	19.97	393,573	17,928	2814
Fitness	226	2,936,618	20.1	369,999	24,589	2132
Influencer	230	2,097,050	25	252,543	12,245	1588
Physician	207	2,280,350	57	159,983	8222	800
Physiotherapist	216	1,583,240	34	154,097	7905	768
Other	196	1,608,473	34	165,549	6313	786.5

TikTok creator identities in the final data set

We coded the 157 TikTok videos in the final data set into six creator identities (Table 6). Chiropractors were the most common identity creating TikTok videos in the final data set (46%; n=72), followed by fitness professionals (22%; n=34), physicians (3%; n=5), and physiotherapists (3%; n=5). In summary, the highest proportion of the final data set was created by chiropractors (Table 3).

**Table 6:** Author identity in the final data set

Author identity	Number of TikTok videos	Percentage of TikTok videos
Chiropractic	72	46%
Fitness professional	34	22%
Influencer	30	19%
Physician	5	3%
Physiotherapist	5	3%
Other	11	7%
TOTAL	157	100%

Intervention themes in the final data set

We examined TikTok videos in the final data from the perspective of three types of intervention: education, exercises, or treatment (Table 7). The salient findings were: (1) videos by fitness professionals (71%; n=24) and chiropractics (63%; n=45) primarily featured exercise content; these TikTok videos generally offered a single exercise, with no information about frequency or duration, (2) videos by influencers primarily featured educational content (73%; n=22); these TikTok videos often featured suggestions about managing ANSBP throughout activities of daily living. The remaining creator identities were represented by a small number of TikTok videos, in which we could not identify a specific theme.

**Table 7:** Intervention themes in the final data set

Author category	1. Education (n=42)	2. Exercise (n=83)	3. Treatment (n=32)	Total (n=157)
Chiropractic	n=7 (10%)	n=45 (63%)	n=20 (28%)	n=72 (100%)
Fitness professional	n=10 (29%)	n=24 (71%)	n=0 (0%)	n=34 (100%)
Influencer	n=22 (73%)	n=5 (17%)	n=3 (10%)	n=30 (100%)
Physician	n=1 (20%)	n=2 (40%)	n=2 (40%)	n=5 (100%)
Physiotherapist	n=1 (20%)	n=3 (60%)	n=1 (20%)	n=5 (100%)
Other	n=1 (9%)	n=4 (36%)	n=6 (55%)	n=11 (100%)

Characteristics of the final data set based on a reference checklist

We analysed the final data set based on the reference checklist. We found differences in the self-care advice contained in TikTok videos based on creator identity (Table 8). Most notably, few videos under any creator identity carried any information about when to visit a medical professional, or red flags indicating potentially serious underlying pathology. Other salient findings were: (1) physiotherapy TikTok videos were most suitable for individuals in acute pain (100%; n=5), most frequently discussed analgesia (60%; n=3), and most frequently provided an appropriate prognosis (60%; n=3); however, the overall number of physiotherapist TikTok videos was small, and this result should be interpreted with caution, and (2) influencer created TikTok videos focused on personal experiences of pain resulting from activities of daily living (70%; n=21) and were most likely to feature an affective dimension (27%; n=8) than other creator identities. Finally, the data in Table 8 is nominal data and is not suitable for statistical analysis.

**Table 8:** Reference checklist results

Creatoridentity	1. Acute (n=104)	2. ADL (n=49)	3. Analgesia (n=17)	4. Red Flag (n=3)	5. Affect (n=10)	6. Appropriate prognosis (n=19)
Chiropractic	n=45 (63%)	n=14 (19%)	n=4 (6%)	n=2 (3%)	n=1 (1%)	n=4 (7%)
Fitness professional	n=17 (50%)	n=11 (32%)	n=2 (6%)	n=1 (3%)	n=0 (0%)	n=9 (26%)
Influencer	n=22 (73%)	n=21 (70%)	n=7 (23%)	n=0 (0%)	n=8 (27%)	n=3 (10%)
Physician	n=4 (40%)	n=1 (20%)	n=1 (20%)	n=0 (0%)	n=0 (0%)	n=0 (0%)
Physiotherapist	n=5 (100%)	n=0 (0%)	n=3 (60%)	n=0 (0%)	n=1 (20%)	n=3 (60%)
Other	n=10 (91%)	n=2 (18%)	n=0 (0%)	n=0 (0%)	n=0 (0%)	n=0 (0%)

## Discussion

TikTok is an increasingly popular medium for disseminating short health messages. This exploratory study aimed to evaluate the scope of ANSBP content available on TikTok as of September 2021. The main cohort using TikTok is young and at risk of ANSBP. However, we suggest that the messages reaching young TikTok users overall do not generally reflect the self-care advice described by Foster et al. [35,40]. We found that methods used for the analysis of YouTube videos may also be adapted to the analysis of TikTok videos.

Metadata

The metadata for the final data set revealed that chiropractic TikTok videos were consistently the most viewed, commented, and shared in our data set. Zheluk and Maddock (also studying ANSBP) similarly found chiropractic videos were consistently the most commonly viewed and most commented videos on YouTube [31]. We did not identify peer-reviewed literature describing the popularity of chiropractic on social media. However, we identified grey literature describing the use of TikTok, YouTube, and Instagram as an important marketing channel by the chiropractic profession [43]. Specifically, this literature describes the focus on cracking sounds associated with spinal manipulation as a form of Autonomous Sensory Meridian Response (ASMR). These ASMR videos are explicitly directed at marketing the chiropractic profession [44]. ASMR refers to the sensations elicited in response to a range of sounds including whispering, spinal manipulation, and eating [45]. Further, chiropractic TikTok videos frequently feature young female patients wearing tight or limited clothing. See for example the TikTok video created by chiropractor Dr Alex [46]. In summary, this suggests TikTok is being actively used by the broader chiropractic profession as a marketing channel.

Creator identities

Chiropractors were the main creator identity identified in our study. Chiropractic TikTok videos in our data set were the oldest of all identities, with an average (mean) of 316 days since publication. This suggests chiropractors have been early adopters of TikTok as a medium. Chiropractic TikTok videos were also the most popular. The higher average age of chiropractic TikTok videos may partially account for their higher view counts. Zheluk and Maddock similarly found a high number of ANSBP videos on YouTube were created by chiropractors [31]. Further, in comparison with YouTube, we found a near absence of physiotherapists (3%; n=5), physicians (3%; n=5), and no videos from yoga instructors (0%) in the final data set. The absence of physiotherapists, physicians, and yoga instructors in #backpain TikTok videos is noteworthy. Zheluk and Maddock suggested video content created by these three disciplines was more consistently aligned with ALBP self-management guidelines than those created by chiropractors or fitness professionals. 

Intervention themes

We examined education, exercise, and treatment as potential intervention themes. The most common intervention theme in our data set was exercise. The limited information about duration, frequency, progression of exercises, and precautions suggest that individuals using this information may be more likely to gain little benefit or risk further pain and injury from conducting these exercises. The short mean duration of TikTok videos in our dataset (e.g., 19.97 seconds for chiropractic videos) compared to YouTube ALBP videos may influence content considerations. TikTok videos in our final dataset frequently featured a single activity. Activities included an exercise, a discussion of pain, or chiropractic manipulation. By contrast, YouTube videos generally offer a slower-paced and more comprehensive examination of low back pain topics. Influencers describing pain and activities of daily living were another notable finding. We coded these influencer TikTok videos as education.

Reference checklist

Overall, we found limited concordance between popular chiropractic and fitness TikTok videos and the ANSBP reference checklist. Most notably, few videos carried any information about when to visit a medical professional or any other precautionary information. This suggests that by excluding disclaimer information, the short duration of TikTok videos may expose creators to some medicolegal risk. Most identities similarly demonstrated limited concordance with the reference checklist. However, TikTok offers creators the option of creating three and five-minute videos. We found some of the longer videos offered greater scope for presenting content similar to YouTube. This suggests that TikTok has the potential to offer guideline-concordant content and more complex advice. Conversely, these longer videos may be less appealing to TikTok audiences [47].

Translation of methods from YouTube to TikTok

We believe that methods translated from YouTube, as described in this paper, may also be appropriate for the analysis of ANSBP video content on TikTok. Specifically, the methods that may be applied to TikTok videos include: (1) the analysis by creator identity to analyse TikTok metadata. The constantly changing popularity of individual TikTok videos suggests individual videos may not offer the optimal unit of analysis. We suggest an analysis of data sets aggregated by creator identity may provide a more consistent approach to the analysis of broader trends in TikTok health information; (2) The analysis of TikTok videos by intervention themes such as education, treatment, and exercise, in combination with creator identity and metadata appears to offer insights into the scope of health information available beyond simple description. These themes should be grounded themes, i.e., they should be based on analysis of the specific data set; and (3) the use of a reference checklist to assess TikTok videos against consensus guidelines as an approach to evaluating ANSBP content across social media.

Limitations

This paper had several limitations. First, we did not examine the literature on digital health interventions. This study examined the scope of ANSBP TikTok videos available in 2021. By conducting this study we also aimed to extend the methods used for the analysis of TikTok videos. Second, we did not examine TikTok videos from the perspective of misinformation. Suarez and colleagues define health misinformation as a “health-related claim that is based on anecdotal evidence, false, or misleading owing to the lack of existing scientific knowledge” [48]. In this study, we identified the use of misleading TikTok videos for marketing purposes by chiropractors. We suggest these TikTok videos appeared motivated by financial gain rather than lack of scientific knowledge and are thus not consistent with this definition of heath disinformation. While the presentation of misleading ANSBP information on TikTok merits consideration, this is beyond the scope of this study.

Further research

In this paper, we made limited comparisons between YouTube and TikTok from the perspective of translating methods for analysing videos across social media platforms. We believe further research that examines the optimal use of the unique features of each popular social media platform by various health disciplines is merited.

Second, there appear to be several opportunities for health professionals seeking to use TikTok for the dissemination of ANSBP information. We identified a potential demand for information about ADL based on TikTok videos produced by influencers. We suggest that the limited presence of mainstream health professionals such as physicians and physiotherapists offer opportunities to reach younger age cohorts with health messages about ANSBP and other health conditions. The use of TikTok by younger age cohorts to self-care and self-manage ANSBP thus also merits further research.

Third, we believe chiropractic TikTok videos merit further research. Chiropractic TikTok videos featuring young women and ASMR represent a novel health marketing phenomenon. We found TikTok videos created by chiropractors to be consistently rated poorly by coders against the ANSBP reference checklist. This marketing phenomenon may be influencing future expectations of what constitutes appropriate ANSBP care among younger TikTok users. 

Fourth, content analysis cannot examine the actual impact of TikTok videos on individual decision making. Future research may establish this causal relationship. In summary, TikTok is a popular social medium that is under-researched. This paper contributes to the TikTok methods literature and to the understanding of the scope of ANSBP information available on TikTok.

## Conclusions

TikTok is a popular social media channel among young people and it is plausible that it may influence decision making about what constitutes appropriate ANSBP self-care in young people. However, we found that most messages reaching younger people about ANSBP self-care do not reflect evidence-informed guidelines. The most viewed TikTok videos about ANSBP are not produced by mainstream health professionals. There is scope for mainstream health professionals to provide evidence-informed self-management and self-care content for ANSBP. This requires that mainstream health professionals adopt creative approaches to transmit their ideas effectively via the novel TikTok social media platform.

## Appendices

**Table 9:** TikTok videos in final data set

NUMBER	CHANNEL	URL	DISCIPLINE
	Chiropractic TikToks		
1	💫TheOsteoDr💫	https://www.tiktok.com/@theosteodr/video/6856464078626360582	Chiropractor
2	Aaron Kubal, DC	https://www.tiktok.com/@aaron_kubaldc/video/6947867891475811590	Chiropractor
3	Aliya Visram	https://www.tiktok.com/@draliyavisram/video/6914851658455436550	Chiropractor
4	BethaltoBackDoc	https://www.tiktok.com/@dr.backcrack/video/6938506940486110469	Chiropractor
5	Daniel DeLucchi DC	https://www.tiktok.com/@chiroseattle/video/6949706466429275398	Chiropractor
6	Daniel DeLucchi DC	https://www.tiktok.com/@chiroseattle/video/6884378171639106822	Chiropractor
7	Daniel DeLucchi DC	https://www.tiktok.com/@chiroseattle/video/6903914794022128902	Chiropractor
8	Daniel DeLucchi DC	https://www.tiktok.com/@chiroseattle/video/6950423971397455109	Chiropractor
9	Daniel DeLucchi DC	https://www.tiktok.com/@chiroseattle/video/6958219855187217670	Chiropractor
10	domenic	https://www.tiktok.com/@domeniciniguez/video/6973420000283479301	Chiropractor
11	Dr Ali Elahi	https://www.tiktok.com/@drelahi/video/6984174061165333766	Chiropractor
12	Dr Donovan Smolich	https://www.tiktok.com/@drdonovan/video/6939957801770880262	Chiropractor
13	Dr Matt Pennell	https://www.tiktok.com/@drmattpennell/video/6770810539246472453	Chiropractor
14	Dr. Alex	https://www.tiktok.com/@occhiropractor/video/6825697649841245445	Chiropractor
15	Dr. Alex Tauberg DC CSCS	https://www.tiktok.com/@thepittsburghchiro/video/6826723541837270278	Chiropractor
16	Dr. Alyssa Hickey	https://www.tiktok.com/@dr.alyssanhickey/video/6816490019193359622	Chiropractor
17	Dr. Anthony Vuckovich	https://www.tiktok.com/@victorychiropractic/video/6924078421848968449	Chiropractor
18	Dr. Ben	https://www.tiktok.com/@drbenhorning/video/6880650101769915653	Chiropractor
19	Dr. Brenda Mondragon	https://www.tiktok.com/@brenda_mondragon/video/6917670476877679877	Chiropractor
20	Dr. Brenda Mondragon	https://www.tiktok.com/@brenda_mondragon/video/6992283351545679110	Chiropractor
21	Dr. Brenda Mondragon	https://www.tiktok.com/@brenda_mondragon/video/6921826675038620934	Chiropractor
22	Dr. Brian Meenan DC	https://www.tiktok.com/@pittsburgh_chiropractor/video/6814477599268490502	Chiropractor
23	Dr. Brian Meenan DC	https://www.tiktok.com/@pittsburgh_chiropractor/video/6816849134792854789	Chiropractor
24	Dr. Chris Cooper	https://www.tiktok.com/@drchriscooper/video/6748845723309067526	Chiropractor
25	Dr. Cracks	https://www.tiktok.com/@dr.cracks/video/6818332046809746693	Chiropractor
26	Dr. Cracks	https://www.tiktok.com/@dr.cracks/video/6919575028187090181	Chiropractor
27	Dr. Cracks	https://www.tiktok.com/@dr.cracks/video/6840091379847138565	Chiropractor
28	Dr. Cracks	https://www.tiktok.com/@dr.cracks/video/6865288520425114885	Chiropractor
29	Dr. Cracks	https://www.tiktok.com/@dr.cracks/video/6915157542670716165	Chiropractor
30	Dr. Cracks	https://www.tiktok.com/@dr.cracks/video/6868253899791289606	Chiropractor
31	Dr. Cracks	https://www.tiktok.com/@dr.cracks/video/6847470009623547142	Chiropractor
32	Dr. Cracks	https://www.tiktok.com/@dr.cracks/video/7001186897951345925	Chiropractor
33	Dr. Cracks	https://www.tiktok.com/@dr.cracks/video/6821539509755235590	Chiropractor
34	Dr. Cracks	https://www.tiktok.com/@dr.cracks/video/6830510490297306373	Chiropractor
35	Dr. Cracks	https://www.tiktok.com/@dr.cracks/video/6826857573820501253	Chiropractor
36	Dr. Grant Elliott	https://www.tiktok.com/@rehabfix/video/6958132865330711814	Chiropractor
37	Dr. Jimmy Sayegh	https://www.tiktok.com/@kingofcracks/video/6916163209174977798	Chiropractor
38	Dr. Jimmy Sayegh	https://www.tiktok.com/@kingofcracks/video/6850477410568162566	Chiropractor
39	Dr. Jimmy Sayegh	https://www.tiktok.com/@kingofcracks/video/6915050096476294406	Chiropractor
40	Dr. Jimmy Sayegh	https://www.tiktok.com/@kingofcracks/video/6944357238576336134	Chiropractor
41	Dr. Jimmy Sayegh	https://www.tiktok.com/@kingofcracks/video/6858026719215947013	Chiropractor
42	Dr. Jimmy Sayegh	https://www.tiktok.com/@kingofcracks/video/6982574991497809157	Chiropractor
43	Dr. Kyle Naylor	https://www.tiktok.com/@drkylenaylor/video/6856919824900099334	Chiropractor
44	Dr. Mike	https://www.tiktok.com/@drmichaelvan/video/6984856294297554181	Chiropractor
45	Dr. Patrick Karamkhodian, DC	https://www.tiktok.com/@doctorkaramkhodian/video/6929923797919223045	Chiropractor
46	Dr. Rashad Trabulsi	https://www.tiktok.com/@dr.rashad.trabulsi/video/6956288693585939714	Chiropractor
47	Dr. Remix	https://www.tiktok.com/@dr.remix/video/6862145627661290758	Chiropractor
48	Dr. Remix	https://www.tiktok.com/@dr.remix/video/6905199614635347205	Chiropractor
49	Dr. Remix	https://www.tiktok.com/@dr.remix/video/6824603797994736902	Chiropractor
50	Dr. Remix	https://www.tiktok.com/@dr.remix/video/6954533716525911302	Chiropractor
51	Dr. Remix	https://www.tiktok.com/@dr.remix/video/6923413529647172870	Chiropractor
52	Dr. Remix	https://www.tiktok.com/@dr.remix/video/6953806031390625029	Chiropractor
53	Dr. Remix	https://www.tiktok.com/@dr.remix/video/6850611478697872646	Chiropractor
54	Dr. Remix	https://www.tiktok.com/@dr.remix/video/6995376258909605125	Chiropractor
55	Dr. Remix	https://www.tiktok.com/@dr.remix/video/6946368828314094854	Chiropractor
56	Dr. Remix	https://www.tiktok.com/@dr.remix/video/6980145907052317958	Chiropractor
57	Dr. Remix	https://www.tiktok.com/@dr.remix/video/6977544868855893253	Chiropractor
58	Dr. Remix	https://www.tiktok.com/@dr.remix/video/6940814731645160710	Chiropractor
59	Dr. Remix	https://www.tiktok.com/@dr.remix/video/6828382067546492166	Chiropractor
60	Dr. Remix	https://www.tiktok.com/@dr.remix/video/6872201834849766662	Chiropractor
61	Dr. Remix	https://www.tiktok.com/@dr.remix/video/6982391971998469381	Chiropractor
62	Dr. Remix	https://www.tiktok.com/@dr.remix/video/6823143616030969094	Chiropractor
63	DrJustinDC	https://www.tiktok.com/@weberspineandjoint/video/6840112110232521989	Chiropractor
64	Efecto Bienestar Quiropráctica	https://www.tiktok.com/@efecto.bienestar.quiro/video/6856437456120679686	Chiropractor
65	Erdal Arsu	https://www.tiktok.com/@erdalarsu/video/6955775187526765829	Chiropractor
66	Josh Adams	https://www.tiktok.com/@drjoshadams/video/6992338421100383494	Chiropractor
67	Manhattan Wellness Group	https://www.tiktok.com/@manhattanwellnessgroup/video/6950357114716048641	Chiropractor
68	Michael Oakson	https://www.tiktok.com/@michael.oakson/video/6843217189844045061	Chiropractor
69	Michael Oakson	https://www.tiktok.com/@michael.oakson/video/6871321368173104389	Chiropractor
70	Michael Oakson	https://www.tiktok.com/@michael.oakson/video/6873586830076300550	Chiropractor
71	Norwood Chiropractic	https://www.tiktok.com/@norwood.chiro/video/6974726857698708742	Chiropractor
72	Root Cause Medical Clinic	https://www.tiktok.com/@rootcausemedicalclinic/video/6927734829375098118	Chiropractor
	FITNESS TIKTOKS		
1	Jake Abela	https://www.tiktok.com/@jake_abelaa/video/6980612856567926018	Fitness
2	emma	https://www.tiktok.com/@endometriosisem/video/6963355040425741574	Firtness
3	Stella Yang	https://www.tiktok.com/@stellatoday/video/6891820204439964929	Fitiness
4	Arun Gray	https://www.tiktok.com/@aginjuryrehab/video/6893188478402612482	Fitness
5	Beef patty	https://www.tiktok.com/@leanbeefpatty/video/6959544199758859526	Fitness
6	Bones to Bulk	https://www.tiktok.com/@bonestobulk/video/6771573634193476870	Fitness
7	cassey	https://www.tiktok.com/@blogilates/video/6884603363997043974	Fitness
8	exercise & food	https://www.tiktok.com/@beautifuldrinks/video/6951328015607532806	Fitness
9	Fitness¦At Home¦YogibeastV2 🔥	https://www.tiktok.com/@fitness_yogibeast_v2/video/6978273324564499718	Fitness
10	Fitness¦At Home¦YogibeastV2 🔥	https://www.tiktok.com/@fitness_yogibeast_v2/video/6926336472090037510	Fitness
11	Fitness¦At Home¦YogibeastV2 🔥	https://www.tiktok.com/@fitness_yogibeast_v2/video/6977902577090399493	Fitness
12	Fitness¦At Home¦YogibeastV2 🔥	https://www.tiktok.com/@fitness_yogibeast_v2/video/6944133830341184774	Fitness
13	Fitness¦At Home¦YogibeastV2 🔥	https://www.tiktok.com/@fitness_yogibeast_v2/video/6901844162648263942	Fitness
14	Fitness¦At Home¦YogibeastV2 🔥	https://www.tiktok.com/@fitness_yogibeast_v2/video/6988293989984144645	Fitness
15	Fitness¦At Home¦YogibeastV2 🔥	https://www.tiktok.com/@fitness_yogibeast_v2/video/6927078647601925382	Fitness
16	Fitness¦At Home¦YogibeastV2 🔥	https://www.tiktok.com/@fitness_yogibeast_v2/video/6964919544225762566	Fitness
17	Fitness¦At Home¦YogibeastV2 🔥	https://www.tiktok.com/@fitness_yogibeast_v2/video/6977532070344084742	Fitness
18	Fitness¦At Home¦YogibeastV2 🔥	https://www.tiktok.com/@fitness_yogibeast_v2/video/6918172625617341702	Fitness
19	Hybrid Calisthenics	https://www.tiktok.com/@hybridcalisthenics/video/6865113800677330182	Fitness
20	Justin Agustin	https://www.tiktok.com/@justin_agustin/video/6855686911722310917	fitness
21	Meg Iwama | Pain free fam✨	https://www.tiktok.com/@megumi.iwama/video/6931168392258882822	Fitness
22	Meg Iwama | Pain free fam✨	https://www.tiktok.com/@megumi.iwama/video/6917656170513616134	Fitness
23	Mike Bosh PAS	https://www.tiktok.com/@postureguy/video/6938237139683462406	Fitness
24	MovementbyDavid	https://www.tiktok.com/@movementbydavid/video/7000383385109531909	Fitness
25	MV Fitness	https://www.tiktok.com/@mv_fitness_training/video/6975620029022784774	Fitness
26	MV Fitness	https://www.tiktok.com/@mv_fitness_training/video/6975278978890337542	Fitness
27	MV Fitness	https://www.tiktok.com/@mv_fitness_training/video/6991274717499886853	Fitness
28	MV Fitness	https://www.tiktok.com/@mv_fitness_training/video/6993141618505207045	Fitness
29	MV Fitness	https://www.tiktok.com/@mv_fitness_training/video/6976296377559141637	Fitness
30	Stella Yang	https://www.tiktok.com/@stellatoday/video/6902447958776646914	Fitness
31	Stella Yang	https://www.tiktok.com/@stellatoday/video/6904314386177903874	Fitness
32	Stella Yang	https://www.tiktok.com/@stellatoday/video/6902055915327950082	Fitness
33	stormiebrooks_	https://www.tiktok.com/@stormiebrooks_/video/6934839611252395269	Fitness
34	Tanish Choudhary	https://www.tiktok.com/@tanish.choudhary_/video/6855364414816750854	Fitness
	Influencer TikToks		
1	Collin	https://www.tiktok.com/@collinurrmom/video/6904448761968266502	Influencer
2	✨Ria✨	https://www.tiktok.com/@__ria9__/video/6884198026718498050	Influencer
3	Abbie Johnson	https://www.tiktok.com/@beanie_333/video/7004616446831594757	Influencer
4	aileen <33	https://www.tiktok.com/@aileen.desireyyy/video/6991338920109821190	Influencer
5	AirwreckEye	https://www.tiktok.com/@airwreckeye/video/6851364197133978886	Influencer
6	angela	https://www.tiktok.com/@angelarobin99/video/6977188309814545670	Influencer
7	baileybitch	https://www.tiktok.com/@..kitty_hello_/video/6965727976973487366	Influencer
8	Christian Romo	https://www.tiktok.com/@iamchristianromo/video/6920015306438020358	Influencer
9	Conscious Mind ®	https://www.tiktok.com/@consciousmind_/video/6984554482185014534	Influencer
10	drayton	https://www.tiktok.com/@draytonpeterson/video/6918213013526891782	Influencer
11	Emily	https://www.tiktok.com/@living.withem/video/6978137923854060806	Influencer
12	Erica💋	https://www.tiktok.com/@am.ericaxo/video/6806751399364381957	Influencer
13	Gary Vaynerchuk	https://www.tiktok.com/@garyvee/video/6758233472445287686	Influencer
14	gracie o	https://www.tiktok.com/@gracieeeeo/video/6841373190736841990	Influencer
15	Juwan Gutierrez	https://www.tiktok.com/@misocolorful/video/6760434395863125254	Influencer
16	Karissa Morman	https://www.tiktok.com/@karissamorman/video/6994530898175708422	Influencer
17	karol	https://www.tiktok.com/@karolscorner/video/7001961826594360582	Influencer
18	Mai	https://www.tiktok.com/@maiifinds/video/6968604276075564294	Influencer
19	malonesedinburgh	https://www.tiktok.com/@malonesedinburgh/video/6993398963571395846	Influencer
20	MEL	https://www.tiktok.com/@mellyd8845/video/6963394207671274757	Influencer
21	Rock Beef	https://www.tiktok.com/@bowenrocky/video/6809482971410681094	Influencer
22	Rosalie	https://www.tiktok.com/@rosaliebodyworks/video/6976405708065557765	Influencer
23	Sarah Spikeston	https://www.tiktok.com/@sarahspikeston/video/6983666941063924997	Influencer
24	The Collective	https://www.tiktok.com/@thecollectiveca/video/7000222327539535109	Influencer
25	The Man	https://www.tiktok.com/@why_does_this_not_work/video/6946692100016622853	Influencer
26	Tik Toker	https://www.tiktok.com/@donkey_meat/video/6992224622183828741	Influencer
27	veronica	https://www.tiktok.com/@veronicagershonn/video/6970983618449526017	Influencer
28	Connor DeWolfe	https://www.tiktok.com/@connordewolfe/video/6962726595589098757	Influencer
29	Kim Kine	https://www.tiktok.com/@kim_kine/video/6908571880819625218	Influencer
30	Stacey Green	https://www.tiktok.com/@staceygreenliving/video/6898779478776974594	Influencer
	Physician TikToks		
1	Dr. Michelle G.	https://www.tiktok.com/@dr.mgmd/video/6831365039916649733	Physician
2	BT Osteopathy	https://www.tiktok.com/@btosteopathy/video/6960850821437295877	Physician
3	BT Osteopathy	https://www.tiktok.com/@btosteopathy/video/6987543766097808645	Physician
4	Stephanie Montrose	https://www.tiktok.com/@stephaniemontrose/video/6984726411055500550	Physician
5	Stephanie Montrose	https://www.tiktok.com/@stephaniemontrose/video/6975328448193531142	Physician
	Physiotherapy TikToks		
1	👨🏽‍⚕️Clayton Dir PT, DPT 🩺	https://www.tiktok.com/@tiktok_physio/video/6959613057517489414	Physiotherapy
2	Dr. Dan, DPT	https://www.tiktok.com/@dr.dan_dpt/video/6831534268599078149	Physiotherapy
3	Mobility MedClinic Inc	https://www.tiktok.com/@mobilitymedclinic/video/7004090059628547334	Physiotherapy
4	👨🏽‍⚕️Clayton Dir PT, DPT 🩺	https://www.tiktok.com/@tiktok_physio/video/6952959974720965893	Physiotherapy
5	Hulst Jepsen Physical Therapy	https://www.tiktok.com/@hulstjepsenpt/video/7001110734247628037	Physiotherapy
	Other TikToks		
1	Tom Loud	https://www.tiktok.com/@tom.loud/video/6822092270725025029	Other
2	MediRestore	https://www.tiktok.com/@medirestore/video/6937474125283773701	Other
3	Sleekform Kneeling Chairs	https://www.tiktok.com/@sleekform/video/7003459089007267078	Other
4	Sleekform Kneeling Chairs	https://www.tiktok.com/@sleekform/video/7003741779656838406	Other
5	Greg Gyuldemirian	https://www.tiktok.com/@greggyuldemirian/video/6885890985876933893	Other
6	Moore Wellness	https://www.tiktok.com/@moorewellness/video/6980850569615248645	Other
7	Moore Wellness	https://www.tiktok.com/@moorewellness/video/6981959221638122758	Other
8	Moore Wellness	https://www.tiktok.com/@moorewellness/video/6988229851190709510	Other
9	Moore Wellness	https://www.tiktok.com/@moorewellness/video/6980113610152463621	Other
10	Reyna	https://www.tiktok.com/@reynacohan/video/6961546204115111173	Other
11	IOHA	https://www.tiktok.com/@instituteofhumananatomy/video/6787103439676558598	Other
